# Barriers and Facilitators for the Donation and Acceptance of Human Breast milk: A Scoping Review

**DOI:** 10.1007/s13668-023-00506-8

**Published:** 2023-11-24

**Authors:** Edlin Glane Mathias, Divya Sussana Patil, Ashwija Kolakemar, Jisha B. Krishnan, Vishnu Renjith, Nachiket Gudi, Ravi Shankar Swamy, Angela Brand

**Affiliations:** 1 Department of Health Information, Prasanna School of Public Health, Manipal Academy of Higher Education, Manipal, India; 2 Department of Nephrology, Kasturba Medical College, Manipal Academy of Higher Education, Manipal, India; 3 School of Nursing and Midwifery, Royal College of Surgeons in Ireland, Dublin, Ireland; 4 Faculty of Health Medicine and Life Sciences (FHML), Maastricht University, P.O. Box 616, 6200 MD Maastricht, The Netherlands; 5 Imperial College Healthcare NHS Trust, London, UK; 6 Manipal Hospitals Bengaluru, Bangalore, Karnataka India; 7 United Nations University – Maastricht Economic and Social Research Institute on Innovation and Technology (UNU-MERIT), Boschstraat 24, NL - 6211 AX Maastricht, The Netherlands; 8 Department of Health Information, Prasanna School of Public Health, Manipal Academy of Higher Education, Manipal, India

**Keywords:** Breastfeeding, Breast milk, Donation, Acceptance, Milk bank, Newborn, Infant

## Abstract

**Purpose of Review:**

Human milk is the best source of nutrients for all infants. When a mother’s own milk is unavailable, the World Health Organization suggests using donor human milk for premature neonates with or without medical complications. Exploring the barriers and facilitators for breast milk donation and its acceptability is essential for developing this intervention. A scoping review was conducted based on a methodological framework developed by Arksey and O’Malley (Int J Soc Res Methodol 8:19–32, 2005). A search was conducted in PubMed (NCBI), CINAHL (EBSCO), and Web of Science (Elsevier). A two-stage sequential screening process was adopted. Data extraction was done using a piloted data extraction form.

**Recent Findings:**

We included 20 articles for narrative synthesis. Barriers and facilitators for donating and accepting breast milk were categorized under six themes: individual, family, community, workplace, health system, and policy-related. The common individual barriers were time requirements for BMD, personal dislike of the process, lack of knowledge, insufficient milk, negative opinions, and lack of information. Family stigma, negative rumors, less educated family members, and illness of a family member were identified as family-related barriers. Community-related barriers include cultural or religious unacceptable practices, societal taboos, and distance to milk banks. The major barriers identified in relation to the health system were lack of practical and psychological support, lack of information, storing and transportation issues, lack of knowledge among HCWs, and logistical challenges of creating a milk lab. The common work-related barriers were the lack of adequate time, philosophical objections, and incomprehension at returning to work. Policy-related barriers identified include the need for hygiene requirements, donation costs, and lack of standardized guidelines.

**Summary:**

Making the donation process faster, providing pick-up services for donors, and community education and male partner engagement regarding breast milk donation could help to boost the acceptability of breast milk donation.

**Supplementary Information:**

The online version contains supplementary material available at 10.1007/s13668-023-00506-8.

## Introduction

Breastfeeding (BF) for at least 2 years is the best method recommended by the World Health Organization (WHO) to provide healthy growth and development to infants. It further recommends BF to be exclusive, where “the infant will have only breastmilk and no other liquids or solids, not even water, for the first six months of life” [1]. Growth hormones, nutrients, enzymes, and immune factors are present in breast milk to ensure a child’s growth and development [2]. Owing to factors such as hypogalactorrhea, lactation insufficiency, agalactia, busy work schedule, lack of knowledge, insufficient milk syndrome, hypogalactia, agalactorrhea, and socio-cultural factors, a mother may be unable to feed the infant [3]. We conclude this gave rise to the formula food industry, reducing global breastfeeding rates. Mother milk bank (MMB) or human milk bank (HMB) [4] has a long-standing popularity. However, the penetration of the formula food market may have greatly contributed to reducing their utility.

*MMB* “is a facility that allows the collection and distribution of human milk donated by lactating mothers other than the biological mother.” Several guidelines and milk banks have been established across Europe over the past few decades that serve as sources of nutrition for infants in need [5]. In a joint statement along with the United Nations International Children’s Emergency Fund (UNICEF) through the Baby-Friendly Hospital Initiative in 1980, the WHO communicated that “*Donated human milk (DHM)* is the desirable alternative when a mother’s milk is not available” [6]. Initially, the “wet nursing method” was used to address breast milk shortage, where a woman breastfeeds another’s child. Later, in 1989, “formal milk banking,” which serves as a repository of donated milk to use when needed, was introduced to use pasteurized donor human milk (PDHM) as a standard [7]. There are about 500 human milk banks across the globe, with over 200 located in Europe and the USA, 70 in Africa, 44 in Asia, and four in Australia. In the African continent, South Africa has 60, Cameroon has six, Kenya has one, and Nigeria has one [8].

Existing literature provided insights on strengthening human milk banking services to decrease neonatal mortality [9, 10]. There are multiple individual studies on the barriers and facilitators for BM donation and acceptance, whereas synthesized evidence is limited [3, 11, 12•, 13]. With the political commitment towards scaling the establishment of milk banks across the countries, it becomes imperative to identify the barriers and facilitators for accepting breast milk donation. This review aims to identify the barriers and facilitators for breast milk donation and acceptance among mothers globally. Based on the findings of this review, advocacy for the donation of breast milk will be made for future breast milk banks, and improved breastfeeding practices will be implemented.

## Methods

Using a scoping review methodology, we explored related literature to gain a deeper understanding of the barriers, facilitators, and acceptability of maternal breast milk donation. This scoping review is reported according to the “Preferred Reporting Items for Systematic Reviews and Meta-Analyses (PRISMA) Extension for Scoping Reviews Checklist” [14]. We adopted the six-stage methodological framework of scoping review by Arksey and O’Malley [15]. The framework consists of the following steps:

### Step 1: Specify the Research Question

1. What are the barriers to and facilitators for breast milk donation?
2. What are the barriers and facilitators for accepting the breast milk of another mother?

We followed the Population, Concept, Context, and Study design criteria for identifying the studies.

#### Population

We included studies conducted on mothers of all age groups and ethnicities. The studies on mothers with lactation insufficiency, insufficient milk syndrome, agalactia, agalactorrhea, hypogalactia, or hypogalactorrhea were included. Studies conducted on families/caregivers who cared for a breastfeeding child or infant with a history of maternal mortality were considered. Studies that restricted mothers from breastfeeding their children to prevent transmission of infectious pathogens were considered.

#### Concept

Breastfeeding (donated human breast milk). The definition of breastfeeding is “the process of feeding human breast milk to an infant, either directly from the breast or by expressing (pumping out) the milk from the breast and bottle-feeding it to the infant” [16]. In the first months of life, breastmilk supplies all the nutrients and energy needed by an infant, and it continues to provide up to half of a child’s nutritional needs during the second half of the first year and up to one-third in the second year [2]. In this review, the human milk bank, breast milk bank, or lactarium is “a service that collects, screens, processes, and dispenses by prescription human milk donated by nursing mothers who are not biologically related to the recipient infant.”

#### Context

The available literature provided inconsistency in the findings. Hence, to understand the barriers and facilitators for breast milk donation and acceptance worldwide, we included studies conducted globally (low, middle, and high-income countries) in hospitals, homes, daycare centers, and nursing homes, irrespective of geographical boundaries.

#### Study Designs

We included studies reporting barriers and facilitators for breast milk donation and acceptance. Quantitative studies, irrespective of designs (observational, cross-sectional, cohort), qualitative, and mixed method studies, were included. Protocols, editorials, social media posts, and magazine reports were excluded as they do not offer substantial information to synthesize. We excluded records where full texts were not available. Only English language literature was included.

### Step 2: Identify the Relevant Literature

A comprehensive search strategy was developed by identifying keywords from the Medical Subject Heading Library (MeSH) browser [17] and discussion with subject-matter experts (RS, AB) and by identifying relevant reviews. To identify all possible sources, four electronic databases, including PubMed (NCBI), CINAHL (EBSCO), Web of Science (Clarivate), and Embase (Elsevier), were searched on 07/12/2022 by EGM and further validated by NG. A polyglot search translator tool was used for translating search strings from PubMed (NCBI) across multiple databases [18]. The search was combined using the Boolean operators such as “AND” and “OR.” Studies published between January 2000 and December 2022 were considered for this review. A detailed search strategy is given in Appendix 1.

### Step 3: Selection of Studies

Results from the database searches were imported into Rayyan software [19]. On de-duplication, title and abstract screening was carried out independently by two reviewers (EM and AJ). The discrepancy in selection was resolved through a consensus-building process. The two reviewers (EM and AJ) then independently reviewed full texts. In case of disagreements, it was resolved through a consensus-building approach (discussing the study and reaching a conclusion) and in consultation with NG. Assessment of the quality of included studies and risk of bias was not conducted as this scoping review aimed to provide an overview of the literature. Therefore, the quality of the included studies will not influence the results and their interpretation.

### Step 4: Charting the Data

Four reviewers independently extracted data independently (EM, AJ, DSP, JK). The data quality was ensured by cross-checking the extracted data by two reviewers (DSP and JK). Relevant data on country/region, sample characteristics, study designs, barriers, and facilitators to breast milk donation and acceptance were extracted using a predesigned data extraction form on Microsoft Excel.

### Step 5: Collecting, Summarizing, and Reporting Results

A narrative approach was used to summarize the findings aided by tables where appropriate. The results are presented with the study characteristics (settings and design), barriers for breast milk donation (individual, social, and systemic), facilitators for breast milk donation (individual, social, and systemic), barriers to accepting DHM (individual, social, and systemic), and facilitators to accept DHM (individual, social, and systemic).

### Step 6: Stakeholder Consultation

We did not conduct stakeholder consultation owing to time and financial constraints.

## Results

**Fig. 1:**
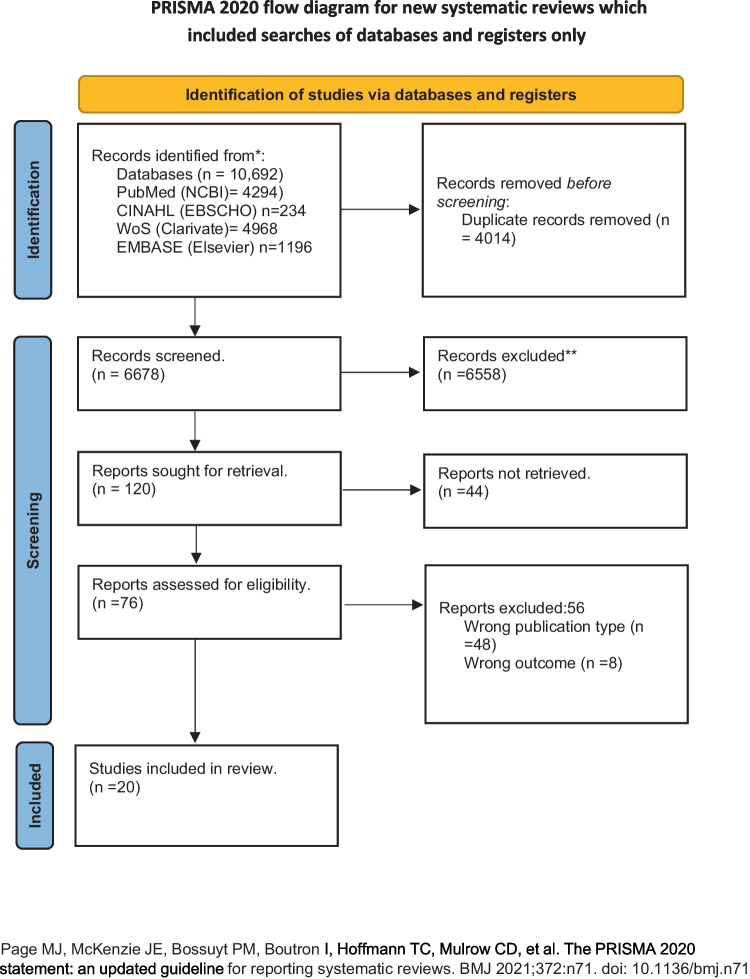
PRISMA flow diagram

Electronic searches were conducted on Medline PubMed (NCBI) (*n* = 4294), Web of Science (Clarivate) (*n* = 4968), EBSCO (CINAHL) (*n* = 234), EMBASE (Elsevier) (*n* = 1196). Of the 10,692 records retrieved, 4014 duplicates were removed using Rayyan. Further, 6678 articles were screened for Title-Abstract, and 76 articles were found eligible for full-text screening. Of the 76 articles, 20 were included for analysis, and others were excluded due to wrong publication type (*n* = 48) and wrong outcome (*n* = 8). The PRISMA flow diagram is presented in Fig. 1. Appendix 2 presents a list of records excluded during the full-text stage.

## Characteristics of Included Studies

### Study Settings

Five studies were conducted in the United States of America (USA) [20–24], three studies were conducted in Turkey [25–27], and three studies were conducted in more than one country: North America, Europe, Oceania, and Asia [28], USA and Canada [29], Australia, Canada, Malaysia, New Zealand, and the USA [30]. Two studies were conducted in India [3, 31•, 32, 33]. One study each was conducted in Sweden [34], Kenya [13], Uganda [12•], Indonesia [35], and UK [36].

### Study Designs

Data to collate evidence on barriers and facilitators for breast milk donation and acceptance was abstracted from mixed methods studies (*n* = 7) [3, 13, 22, 25, 34–36]; descriptive cross-sectional surveys (*n* = 7) [20, 21, 26–28, 30, 32]; qualitative studies (*n* = 3) [12•, 29, 31•] *n* = 1) [33]. Two research articles [23, 24] did not report the study design. The characteristics of the included studies are detailed in Table 1.

**Table 1:** Characteristics of the included studies

**S. no**	**Study ID**	**Study design**	**Geographic location**	**Setting**	**Population**	**Objective**	**Outcome**
1	Olsson et al. [34]	A prospective, mixed method study (Survey was done through mail)	Sweden	Health facility (two referral university hospital milk banks)	Human milk donors (*N* = 72) at two Swedish hospitals.	To describe Swedish human milk donor’s experience during the donation process.	The donors wanted to help other infants, and they had more milk than they needed, they knew women whose infants needed donated human milk, and were aware of the importance of human milk for young children.
2	Schafer et al. [21]	An online cross‐sectional survey (Descriptive cross-sectional survey)	USA	Community	206 participants from the USA classified as milk sharing recipient.	To explore factors associated with emotional responses to a parent’s decision to feed their infant with shared human milk.	Receiving strong spousal/partner support for milk sharing (*p* < .001) and screening donors regarding the health of their nursling(s) (*p* < .05) were associated with more positive emotional responses. Social stigmatization of milk sharing may negatively influence emotional responses among recipient mothers.
3	Kimani-Murage et al. [13]	Mixed methods study	Kenya	Both the health facility and community levels.	Mothers with children younger than 3 years were interviewed using the structured interviews. How was quantitative data collected?	To establish the perceptions on donating and using DHM and establishing human milk banks (HMBs) as Kenya has not yet established HMBs for provision of safe DHM, which is free from any physical, chemical, microbiological contaminants, or pathogens.	Majority of them had a positive attitude towards donating breast milk to a HMB (80%) and feeding children on DHM (87%). At a personal level, participants were more willing to donate their milk to HMBs (78%) than using DHM for their own children (59%).
4	Wambach et al. [20]	Descriptive cross-sectional study	North America	Community	A convenience sample of 50 women who were current or past milk donors within the last year at a regional milk bank participated.	To describe the personal and social aspects of mothers’ milk donation to a milk bank in the Midwest USA.	Barriers were related to process of expression and donation, dealing with milk donation rules and restrictions. Some fathers had concerns about there being enough milk for their own infant, and some needed information and education about the process and purpose of milk donation.
5	Biggs et al. [32]	Prospective, cross-sectional study (Descriptive cross-sectional survey)	South Africa	Community	Mothers (*N* = 37) were interviewed using a telephone-administered questionnaire.	To determine why mothers who had committed to donating to a human milk bank in South Africa did not donate their milk.	The major barrier was infrastructure-related, as 62.2% were unaware of the process after discharge. This was followed by practical issues including no transport (21.6%), no freezer for milk storage (18.9%), or working (5.4%).
6	Gribble et al. [28]	Descriptive cross-sectional survey	North America, Europe, Oceania, and Asia	Community	Milk donors (*n* = 98) and milk recipients (*n* = 41) who had donated or received breast milk were required to have donated or received breast milk in the previous 6 months in an arrangement that was facilitated via the Internet.	To explore the intersection of peer-to-peer milk sharing and donor milk banks.	One-half of donor recipients could not donate to a milk bank because there were no banks local to them or they did not qualify as donors. Other respondents did not donate to a milk bank because they viewed the process as difficult, had philosophical objections to milk banking, or had a philosophical attraction to peer sharing.
7	McCloskey et al. [29]	Qualitative research (In-depth interviews)	USA and Canada	Community	Mothers (*N* = 20) in the USA and Canada who were recipients of peer-to-peer human milk sharing.	To examine the experiences of mothers who have received donated human milk from a peer.	Challenges to peer-to-peer human milk sharing were (a) substantial effort required to secure human milk; (b) institutional barriers; (c) milk bank–specific barriers; and (d) lack of societal awareness and acceptance of human milk sharing. Facilitators included (a) informed decision-making and transparency and (b) support from healthcare professionals.
8	Magowan et al. [12•]	A qualitative study (Focus group discussions and in-depth interviews)	Uganda	Health facility	Caregivers (*N* = 28) (mother, grandmother, or father) of any neonate currently, or recently, admitted in the NNU and in-depth interviews with four of the eight healthcare workers (HCWs) from the NNU.	To show that donor human milk can be acceptable to the caregivers of vulnerable babies in hospital settings in Uganda.	Lack of knowledge of donated human milk emerged with discussants, and the barriers relating to transmission of infection (HIV) and poor hygiene
9	Varer et al. [25]	Mixed methods study (In-depth interviews)	Aydin	Community	Two seventy-one women who were aged over 18 years and had a birth in the last 5 years in Çeştepe, Aydın, were included in the quantitative study. In-depth interviews were conducted with 33 women	To determine the opinions, knowledge, and attitudes of native Turkish and refugee women living in Çeştepe, Aydın, a rural area in Turkey, about HMB	Most (59.7%) of the women were willing to donate breast milk, whereas only 27.7% were willing to use donor milk for their babies. Religious concerns, fear of infectious diseases, and distrust in people they did not know were among the reasons for the negative attitudes of the women
10	Mondkar et al. [3]	Qualitative research (In-depth interviews)	Mumbai	Health facility	Qualitative research was conducted among 56 service recipients including mothers and key influencers and 9 service providers to understand their perceptions and practices.	To understand the perceptions and acceptability of DHM and HMB among service providers, individual mothers availing services and influencers.	Challenges shared were limited supply of DHM because of low awareness on milk donation, shortage of trained staff, and risk of milk contamination. Although most mothers were comfortable in donating milk, few were reluctant to donate milk as they feared shortage of milk for their own babies, or milk expression may cause weakness.
11	Lubbe et al. [33]	Observational study (Focus group discussion)	South Africa	Health facility	In total, eight FGDs were conducted: three with mothers (*n* = 13), three with grandmothers (*n* = 17), and two with healthcare professionals (*n* = 11) working with infants.	To determine attitudes of key stakeholders, including mothers, healthcare workers, and grandmothers, regarding the donation and receipt of human breastmilk.	Barriers included the processes for donating and receiving milk, safety, human immunodeficiency virus (HIV) screening, and cultural beliefs. Mothers’ fears included having insufficient milk for their own infants, changes in the quality of donated milk during pasteurization and transportation, and HIV transmission.
12	Murray et al. [35]	A mixed methods study using a convergent parallel design concurrently collecting qualitative and quantitative data (Individual interviews)	Indonesia	Health facility	Mothers (*N* = 74) of hospitalized infants and 8 hospital staff (5 nurse managers, 1 doctor, and 2 administrators) Private, individual qualitative interviews with health professionals, mothers of sick infants completed a paper-based questionnaire with assistance from trained data collectors. Facility observation of the neonatal intensive care unit (NICU), special care unit (SCU), and post-natal and pediatrics wards was iteratively undertaken using a paper-based structured rubric.	This study explored the barriers and enablers to exclusive breastfeeding among sick and low birth weight hospitalized infants in Kupang. The attitudes and cultural beliefs of health workers and mothers regarding the use of donor breast milk (DBM) were also explored.	Mothers (39.7%) retrospectively reported exclusively breastfeeding and 37% of mothers expressed breast milk. Most mothers stated that they would not accept milk from a donor and would use formula if they could not breastfeed. Most mothers stated that they would not accept milk from a donor and would use formula if they could not breastfeed.
13	Perrin et al. [22]	A qualitative design using a grounded theory approach (Semistructured telephone interviews)	USA	Community	Women (*N* = 28)	To explore how lactating women with a surplus of breast milk come to the decision to share their milk with a peer rather than donate to a milk bank.	Participants reported that they received no information about milk exchange options and considerations from healthcare professionals.
14	Osbaldiston et al. [24]	Study design not reported (Telephonic interviews)	Austin	Community	Data were collected via telephonic interview and by sending cover letters and consent forms through mails among 87 mothers.	To create a detailed characterization of human milk donors, including descriptive information about demographics and lifestyle, involvement with the milk bank, reasons for donating, problems encountered while breastfeeding and pumping milk, barriers to donating milk, affective experiences, and personal values.	The reasons for donating milk were significantly correlated with amount of milk donated, namely, “Had too much milk and wanted to donate it”. “Needed to pump milk to stimulate lactation”. A small group of donors reported that they had to pump milk to stimulate lactation. There was a significant negative relationship between age and donation amount such that younger donors donated more milk.
15	Gribble et al. [30]	Descriptive cross-sectional survey design (Closed- and open-ended questions)	Australia [3], Canada [4], Malaysia [2], New Zealand [3], and the USA [28]	Community	Written questionnaire administered to 41 peer milk recipients from five countries.	To examine internet-facilitated peer-to-peer milk sharing, the process by which women came to use peer-shared milk was explored.	Ten respondents considered using peer-shared milk after a friend or relative suggested it; eight had thought about milk sharing prior to needing it; for five respondents, it was viewed as a logical solution or had been suggested by a health worker; for four respondents, online discussions had alerted them to the possibility; three respondents each stated that they had turned to peer-shared milk after failing to obtain banked donor milk or
16	Brown et al. [36]	Mixed methods with thematic analysis (Online questionnaire included both open- and closed-ended questions)	UK	Community	Mothers (*N* = 107) of baby aged 0–12 months who had received screened DHM from a milk bank were administered a Likert scale and open-ended questionnaire.	To explore how experience of receiving DHM for their baby affected the well-being of parents.	Almost all the 107 participants agreed that receiving DHM had a positive impact upon infant health and development, their own mental and physical health, and their family’s well-being. Receiving DHM helped mothers to process some of their emotions at not being able to breastfeed, in part because knowing their baby was being fed gave them the space to focus on recovery and bonding with their baby.
17	Mantri et al. [31•]	Qualitative study (In-depth interview of key stakeholders)	India	Community	A face-to-face interview was conducted using the in-depth interview (IDI) guide for lactating mothers (*n* = 30) and healthcare providers (*n* = 25)	To know the various facets of the challenges in milk banking practices using the Root Cause Analysis framework.	Lack of recurring funds, dedicated lactational counsellors, and trained technicians were the challenges. The community challenges were low acceptance of DHM due to safety concerns, risk of disease transmission, and quality of donated milk. Moreover, the religious stigma and cultural beliefs regarding the transfer of heredity traits and decrease in mother–child affection act as barriers in donating milk.
18	Mizrak Sahin [27]	Descriptive cross-sectional study	Turkey	Health facility	Multipara mothers (*N* = 250)	To evaluate the attitude, knowledge, and views of mothers about breast milk donation and HMBs.	Mothers (40.8%) indicated that they were against the establishment of HMBs in Turkey. However, only 61 mothers (24.4%) approved of obtaining milk from HMBs. Mothers who did not agree to the establishment of HMBs (77.5%) stated that babies who are fed breast milk from the same mother would be milk siblings, and it would be an ethical problem if they got married to each other.
19	Rosenbaum et al. [23]	Article (Design not reported) Telephonic interview	USA	Health facility	NR	To examine how three hospitals have used pasteurized donor milk successfully despite barriers encountered when incorporating this change in practice.	Common strategies that helped drive success included a strong change agent, a multidisciplinary team, and incorporation of the latest research and guidelines. Barriers encountered included cost, difficulty gaining staff buy-in, and the logistical challenge of creating a milk lab.
20	Gürol [26]	Descriptive cross-sectional study (Open-ended and closed questions)	Turkey	Health facility	Married women aged 15–49 years who had given birth and who were registered with a family health center in Turkey.	To determine the knowledge and views of Eastern Anatolian women towards mother’s milk banking.	Most 90.6% indicated they had not previously heard anything about breast milk banking, 64.0% said that they could donate their milk, 36.3% stated it constituted a problem from a religious aspect, and 28.9% said it leads to social and moral problems.

Our review categorized the barriers and facilitators to breast milk donation and acceptance as individual, social, and systemic factors.

- **1. Barriers for breast milk donation**
  - **(a) Individual barriers**
    The scoping review identified multiple factors as barriers to donating breast milk. The most commonly reported barriers were lack of knowledge among mothers regarding donation [20, 22, 31•, 32–34], lack of time due to daily chores [20, 22, 24, 31•, 33, 34], physical and psychological stress from pumping, freezing, or storing milk [3, 24, 31•, 33], cost and distance to the milk bank [20, 22, 24, 28, 31•, 12•, 24, 33]. Other barriers reported by mothers/caregivers were personal dislike by the mothers [13], unable to express breast milk [32], insufficient milk to donate [13, 32], hygiene concerns [13, 34], medical condition of the mother (for example, engorgement, cracked or chapped nipples and breast infection) [24, 28], risk of disease transmission [12•, 13, 27], fear of transfer of genetic traits and familial diseases [13], negative influence affecting the mother–child relationship [13, 33], mother unable to meet the needs of her own child [3, 31•, 33], lack of appropriate storage arrangements [24, 32], difficulty to trust unknown donors [27], perception that the donated milk loses its strength [13], existing practices and perceptions [12•], and concerns about the works involved in becoming a donor such as getting screened, adhering to MB protocol, shipping milk, and altered milk composition during pasteurization [22, 28] as major barriers for donation. There were a few misconceptions, too, such as changes in skin color if the milk is received from another mother [12•] and the possibility of fluctuation in breast size [33]. A few participants refused to donate, assuming that it may make the mothers receiving donated breastmilk irresponsible towards the child [13].
  - **(b) Social barriers**
    The socio-cultural values within the family played a prominent role in breast milk donation [31•]. A study conducted in eastern Uganda highlighted those taboos circulating in the community regarding breast milk donation, and the husband being the decision-maker in the home prevented mothers from breast milk donation [12•]. Furthermore, it was identified that families having a poor educational background [3] and lack of support to the mother from family members in terms of caring for her own child while she donates discouraged mothers from donating breast milk [31•]. Owing to the illness of a family member, mothers reported that they did not find sufficient time for donating breast milk [24]. The influence of community as a barrier to donating breast milk was identified by six studies conducted across Kenya, South Africa, Eastern Uganda, Turkey, and India [12•, 13, 26, 27, 31•, 32]. Various cultural and religious practices and taboos in the community hindered mothers from donating breast milk [13, 26, 27]. Additionally, a lack of affordable transportation to the milk bank in their locality [32], lack of transparency and health education [12•], and cultural myths, taboos, a lack of awareness, and motivation demotivated mothers from donating breast milk [31•]. The review identified only one study where mothers reported difficulty in expressing milk due to lack of time when they were at work, and that prevented them from donating the milk [32].
  - **(c) Systemic barriers**
    Our review identified eight studies that emphasized systemic factors, which acted as barriers to breast milk donation [3, 20, 22, 24, 27, 31•, 34, 35]. Lack of practical and psychological support from the health professionals [22, 34, 35], shortage of trained staff [3], issues in communication with staff [24], improper acquisition and storage process of breast milk [27, 35], lack of infrastructure, technical and financial support to the milk bank [31•], and hospital discouraging donation due to risk of infection [35] were identified as health system-related barriers to donate breast milk. In addition, the review identified that dealing with milk donation rules and restrictions demotivated mothers in the North American region from donating breastmilk [20].
- **2. Facilitators for breast milk donation**
  - **(a) Individual facilitators**
    Eight studies conducted across Sweden, the USA, Turkey, India, Europe, Asia, and Oceania reported various factors enabling mothers to donate breast milk. A strong desire to help infants [20, 24, 28, 31•, 34], personal values such as security, tolerance, self-direction, and social concern [3, 20, 28, 31•, 34], and awareness about breastmilk donation [20, 24, 25, 27] were most commonly reported facilitators to donate breastmilk. Other facilitators reported were abundant milk supply [20, 24], economic compensation and appreciation [34], own baby being healthy and growing well [20], to stimulate lactation [24], publicity about the shortage of breastmilk [28], and reward or appreciation for the donation of breastmilk [31•].
  - **(b) Social facilitators**
    In our review, we identified one study that emphasized on family-related facilitators for breast milk donation. Wambach et al. [20] identified that instrumental support from the spouse/partner, such as physical help with sterilizing equipment and emotional support, motivated mothers to donate breast milk. In addition, the community members believed feeding a child who requires breastmilk was a good deed, which was identified as a community-related facilitator to donate breastmilk [25]. Another study also reported that support from community leaders encouraged mothers to donate breastmilk [31•].
  - **(c) Systemic facilitators**
    Encouragement from healthcare professionals [20], trust in healthcare professionals [25], counselling by pediatrician/lactation counsellor during the ward rounds and at well-baby clinic [3, 31•], and support from community health workers enhance the knowledge levels about milk banking [31•] were considered as facilitating factors to donate breastmilk to the HMB in India.
- **3. Barriers to accept DHM**
  - **(a) Individual barriers**
    Fear of disease transmission/HIV transmission/allergies, transfer of genetic traits, and familial diseases [13, 25, 31•, 33] were the most cited reasons for not accepting DHM. A few studies reported concerns about milk quality/safety, maintaining the cold chain, and transporting donated milk [3, 31•, 33], preventing them from accepting donated milk. Other findings such as perceived hygiene of the donor/donation process [3, 12•, 13, 25, 35], babies had to be fed by their mother [25], negative influence on bonding between mother and child [13, 31•, 13, 31•, 29], supply could not keep up with demand [29], lack of awareness about milk banks [29], concern about insufficient milk [29], worry about collection, storage and fear of spoiled breastmilk [25], lack of trust in people/donated human milk [25, 35], children not meeting requirement criteria [28], travel and cost [23, 28], and concern about the identity (religion/health) of the donor [28, 35] were barriers that prevented mothers from accepting DHM.
  - **(b) Social barriers**
    The scoping review identified negative social and family stigma (exposure to substances in shared milk, health risk) [21], husbands’ decision-making power [12•], lack of awareness among grandmothers [3], safety concerns [3], and family customs and beliefs [31•] as barriers to accept donated breastmilk. Unacceptable religious and cultural practices hindered the acceptance of donated human milk [13, 23]. Lack of support from the community and lack of societal awareness and acceptance were identified in the USA and Canada [29]. In addition, rumors that donated milk from another mother could be poisonous prevented the acceptance of donated human milk [12•]. Another important barrier identified in a few regions was that, according to Islamic culture, children were not allowed to intermarry if they were fed by the same mother [13, 25]. This prevented mothers from using DHM. Only one study highlighted the lack of a paid parental leave policy in the USA as a barrier to accessing DHM donations because it required time and effort, which conflicted with mothers’ need to return to work [29].
  - **(c) Systemic barriers**
    The most reported barriers to accepting donated human milk were logistical barriers to distance, cost, pasteurization of HM, prescription requirements, the need to prioritize HM for sick and preterm infants, and lack of milk lab [23, 29]. Other barriers to accept donated human milk were hospital/profession policy that discouraged the usage of donated milk [29], negative feedback or lack of support from healthcare, lactation, and adoption professionals [29], and the lack of experience/knowledge of the use of HM [12•].
- **4. Facilitators to accept DHM**
  - **(a) Individual facilitators**
    The most cited reason to accept DHM was that it was a logical solution for insufficient milk/preterm baby/LBW baby in NICU [3, 13, 23, 30, 35, 36]. The other reasons to accept DHM were illness of mother/on medication [3, 13, 31•, 36], screening health status of donor’s nursling [21], unavailability of mother to breastfeed [13], lack of trust in infant formula [30], child unable to tolerate formula feed [30], awareness about the importance of BM [30] and donated human milk [29], child not gaining weight [30, 36], lack of informed decision-making and transparency (with respect to personal and medical history) [29, 33], wet nursing only by family member [12•, 33], newborn unable to suck or is sick [31•, 35, 36], and families with orphaned or abandoned child [31•]. In addition, due to the inability to express milk while at the workplace, most mothers opted to accept donated HM [30].
  - **(b) Social facilitators**
    Strong spousal/partner support [3, 21], the suggestion by a friend or relative [30], and husband decision-making power [12•] were facilitators identified concerning the family members for mothers to accept donated milk. The community played an essential role in facilitating the acceptance of donated HM. Educating the community [12•], considering screening to involve testing heritage and genes (culture compatibility) [33], education to community leaders and traditional healers [33], and perceptions that receiving milk from the bank did not lead to religious and ethical problems [26] were facilitating factors that enabled mothers to accept donated HM.
  - **(c) Systemic facilitators**
    The most reported facilitator was the support from healthcare professionals (lactation consultants, midwives, childbirth instructors, doulas, nurses, and physicians) [12•, 23, 25, 29, 30]. Other facilitators that supported mothers to accept DHM were testing and sterilizing equipment to process BM to ensure milk safety [13, 33], explaining the process of screening, pasteurization, and milk banking (sensitization to mothers and community [12•], and affirming that donors were healthy [12•].

## Discussion

The concept of milk banks has been emerging in the twenty-first century, and there are multiple individual studies on the issue. Our scoping review identified the demand and supply side factors influencing the overall nutritional practices in different regions. We synthesized the barriers and facilitators for donating and accepting individual, social, and systemic factors.

The common individual barriers were time requirements for BMD, personal dislike of the process, lack of knowledge, insufficient milk, negative opinions, and lack of information. Family stigma, negative rumors, less educated family members, and illness of a family member were identified as family-related barriers. Community-related barriers include cultural or religious unacceptable practices, societal taboos, and distance to milk banks. The major barriers identified in relation to the health system were lack of practical and psychological support, lack of information, storing and transportation issues, lack of knowledge among HCWs, and logistical challenges of creating a milk lab. The common work-related barriers were the lack of adequate time, philosophical objections, and incomprehension at returning to work. Policy-related barriers identified include the need for hygiene requirements, donation costs, and lack of standardized guidelines.

Doshmangir et al. [37] identified three domains of barriers to milk donation, which include individual, systematic, and social barriers, which aligns with the findings of our current review. Across all studies, a lack of knowledge regarding milk banking is considered a major barrier to milk donation. These findings are consistent with those of Wambach et al. [20] and Biggs et al. [32], and this highlights the need to improve public awareness of the need for educating expecting mothers and their spouses regarding BMD. Family and spouse education could also be meaningful as they play a major role during the post-partum period. Schafer et al. [21] state that strong spousal support for milk sharing is essential.

Additionally, barriers such as a decrease in mother–child affection, religious stigma, cultural beliefs on the transmission of hereditary traits, and reduced mother–child affection impede the utility of these donation systems. This also accords with the observations made by Doshmangir et al. [37] in their synthesis. According to the report by Olsson et al. [34], Swedish women can donate human milk within the first 3 months after delivery. The concept of HMB warrants the active participation of stakeholders, and understanding the socio-cultural context is vital. Liaising with religious groups, involving stakeholders, and inclusive policy-making could be beneficial in dealing with religious barriers to a greater extent.

Ozdemir et al. [38] found that suckling milk from a donor makes her a wet nurse, while Santos et al. [39] synthesized that the donation of breast milk gave the bereaved mothers a special feeling. Brown et al. [36] reported that sharing a mother’s milk reduced stress and symptoms of depression for the non-lactating mother, as DHM protected their baby from illness. This further highlights the importance of the mother’s perception of this practice embedded in a complex socio-cultural context. Murray et al. [35]found that once the recipient gains trust in the storage systems’ nutritional quality and hygiene, they are more likely to opt for the services.

The MMBs are not devoid of challenges. The systemic challenges identified in metropolitan cities in India were limited recurring funds and a lack of trained human resources such as dedicated lactational counselors and trained technicians [31•, 40•, 31•, 41] echo that breastfeeding is a collective responsibility and can be addressed through multilevel and multicomponent interventions across different settings.Our study highlights the importance of capacity building among health professionals as a lack of knowledge among health professionals was identified as a major health system-related challenge. A review by O’Hare et al. [42] highlights the importance of training health professionals in human milk banking. The benefits and utility of HMB must be incorporated into the training programs for front-line health workers and hospital nurses as they are closely interacting with expecting and new mothers. An essential factor that was unnoticed in our scoping reviews was the influence of commercial milk formulas (CMF). To overcome challenges, stakeholders must take responsibility for achieving breastfeeding goals, such as promoting exclusive breastfeeding, reducing the dependence on formula feed, and setting up milk banks [31•].

Less than half of the world’s infants are breastfed despite evidence showing the long-term benefits of breastfeeding [43]. The CMF industry seems to have normalized the use of formula foods not only to the mother’s inability to express breast milk but also to those who can. The unparalleled success of the CMF industry has been attributed to the industry’s marketing and lobbying tactics [44]. Doherty et al. [45] have called for radical transformation to build resilience in breastfeeding. One of the interventions to respond to this call is setting up DMB (donor milk banks) at district-level hospitals. India has been setting up breast milk banks across various district hospitals, and this step’s impact would need to be evaluated [46].

### Strengths and Limitations

There is a dearth of synthesized literature on the donation and acceptance of human breast milk. This review may be one of the first to attempt to uncover this complex socio-cultural phenomenon. We have utilized various electronic databases to search the literature. The studies have been explored in greater detail to shed light on various factors affecting both the donation and acceptance of breast milk. The study offers insights to practitioners, policymakers, researchers, and patient groups. This review has not included gray literature owing to its extensivity, and we acknowledge it as a limitation. Future studies may qualitatively explore the barriers and facilitators for milk sharing among tribal communities, underprivileged societies, and migrants, as their infant population is at a higher risk of undernourishment. Studies may also be conducted to elucidate mothers’ and caregivers’ responses on the influence of commercial milk formulas on the donation/acceptance of breast milk. The need for synthesizing evidence on interventions and policies to promote the donation and acceptance of donated breast milk is required.

## Conclusion

Plans for establishing breast milk banks should include measures to guarantee that the donation and receipt of processes go without delays. Reducing the time spent at the donation center, providing pick-up service to donors, educating the community about breast milk donation, and encouraging male partners to participate could all help to boost acceptance of breast milk donation.

Donating breast milk can be made more acceptable by maximizing donor convenience, providing pick-up services, and educating the community about breast milk donation. Breastfeeding donation and acceptance rates may be increased by establishing laws to govern the HMB and the marketing of formula foods. We can ensure that important health and development goals are met by ensuring good nutrition for the infant.

### Supplementary Information

Below is the link to the electronic supplementary material.

Supplementary file1 (DOCX 27 KB)

## Data Availability

All the data and materials related to the study are presented in the Appendix.
