# Supplementary material for: Barriers and Facilitators for the Donation and Acceptance of Human Breast milk: A Scoping Review
Source: Curr Nutr Rep. 2023 Nov 24;12(4):617–34. doi: 10.1007/s13668-023-00506-8 (PMC10766659; doi:10.1007/s13668-023-00506-8)
Supplement: Supplementary file 1 — Supplementary file1 (DOCX 27 KB) [file 13668_2023_506_MOESM1_ESM.docx]

**Appendix 1**

**Search strategy**

| **Database** | **Search strategy** | **Hits** |
| --- | --- | --- |
| PubMed | (("barrier*"[Text Word] OR "facilitat*"[Text Word] OR "challenge*"[Text Word] OR "success*"[Text Word] OR "constrain*"[Text Word] OR "difficult*"[Text Word] OR "interfer*"[Text Word] OR "obstruct*"[Text Word] OR "problem*"[Text Word] OR "restrain*"[Text Word] OR "restrict*"[Text Word] OR "enabler*"[Text Word])) AND ("milk banks"[MeSH Terms] OR "Breast milk expression"[MeSH Terms] OR "Breast milk expression"[MeSH Terms] OR "Breast milk"[All Fields] OR "Human milk"[All Fields] OR "Human donor breast milk"[All Fields] OR "Milk sharing"[All Fields] OR "Human milk banking"[All Fields] OR "breast milk donor*"[All Fields] OR "human milk donat*"[All Fields] OR "Milk bank"[All Fields] OR "Mother's milk"[All Fields] OR "milk donation"[All Fields] OR "Breast milk expression"[All Fields]) Filters: from 2000 - 2023 | 4,294 |
| CINAHL | ((barrier* OR facilitat* OR challenge* OR success* OR constrain* OR difficult* OR interfer* OR obstruct* OR problem* OR restrain* OR restrict* OR enabler*)) AND ((MH "milk banks+") OR (MH "Breast milk expression+") OR (MH "Breast milk expression+") OR "Breast milk" OR "Human milk" OR "Human donor breast milk" OR "Milk sharing" OR "Human milk banking" OR "breast milk donor*" OR "human milk donat*" OR "Milk bank" OR "Mother's milk" OR "milk donation" OR "Breast milk expression") | 234 |
| EMBASE (Elsevier) | ((barrier* OR facilitat* OR challenge* OR success* OR constrain* OR difficult* OR interfer* OR obstruct* OR problem* OR restrain* OR restrict* OR enabler*)) AND ((MH "milk banks+") OR (MH "Breast milk expression+") OR (MH "Breast milk expression+") OR "Breast milk" OR "Human milk" OR "Human donor breast milk" OR "Milk sharing" OR "Human milk banking" OR "breast milk donor*" OR "human milk donat*" OR "Milk bank" OR "Mother's milk" OR "milk donation" OR "Breast milk expression") | 1,196 |
| WOS | ((barrier* OR facilitat* OR challenge* OR success* OR constrain* OR difficult* OR interfer* OR obstruct* OR problem* OR restrain* OR restrict* OR enabler*)) AND ("milk banks" OR "Breast milk expression" OR "Breast milk expression" OR "Breast milk" OR "Human milk" OR "Human donor breast milk" OR "Milk sharing" OR "Human milk banking" OR "breast milk donor*" OR "human milk donat*" OR "Milk bank" OR "Mother's milk" OR "milk donation" OR "Breast milk expression") | 4,968 |
| Total |  | **10,692** |

**Appendix 2**

**List of excluded studies at full stage**

| **S.No** | **Study Id** | **Reference** | **Reason for exclusion** |
| --- | --- | --- | --- |
| 1 | Underwood M. A. (2013) | Underwood M. A. (2013). Human milk for the premature infant. Pediatric clinics of North America, 60(1), 189–207. https://doi.org/10.1016/j.pcl.2012.09.008 | Wrong publication type |
| 2 | Bertino et al. (2018) | Bertino, E., Peila, C., Cresi, F., Maggiora, E., Sottemano, S., Gazzolo, D., Arslanoglu, S., & Coscia, A. (2018). Donor Human Milk: Effects of Storage and Heat Treatment on Oxidative Stress Markers. Frontiers in pediatrics, 6, 253. https://doi.org/10.3389/fped.2018.00253 | Wrong publication type |
| 3 | Bramer et al. (2021) | Bramer, S., Boyle, R., Weaver, G., & Shenker, N. (2021). Use of donor human milk in nonhospitalized infants: An infant growth study. Maternal & child nutrition, 17(2), e13128. https://doi.org/10.1111/mcn.13128 | Wrong publication type |
| 4 | Zobbi et al. (2021) | Zobbi, V. F., Fumagalli, S., Antolini, L., Bianco, A., Mornatta, M., Colciago, E., & Nespoli, A. (2021). Women's knowledge and attitude towards human milk donation: a survey within the Italian context. Minerva pediatrics, 10.23736/S2724-5276.21.06339-4. Advance online publication. https://doi.org/10.23736/S2724-5276.21.06339-4 | No full text |
| 5 | Vishnu et al. (2018) | Vishnu Bhat, B., & Adhisivam, B. (2018). Human Milk Banking and Challenges in Quality Control. Indian journal of pediatrics, 85(4), 255–256. https://doi.org/10.1007/s12098-018-2635-y | Wrong publication type |
| 6 | Jarmoc et al. (2021) | Jarmoc, G., Bar-Yam, N., Hagadorn, J. I., Tosi, L., & Brownell, E. A. (2021). Demographics and Geographic Distribution of Mothers Donating to a Nonprofit Milk Bank. Breastfeeding medicine: the official journal of the Academy of Breastfeeding Medicine, 16(1), 54–58. https://doi.org/10.1089/bfm.2020.0197 | Wrong publication type |
| 7 | Lording et al. (2006) | Lording R. J. (2006). A review of human milk banking and public health policy in Australia. Breastfeeding review: professional publication of the Nursing Mothers' Association of Australia, 14(3), 21–30. | No full text |
| 8 | Clifford et al. (2022) | Clifford, V., Klein, L. D., Brown, R., Sulfaro, C., Hoad, V., Gosbell, I. B., & Pink, J. (2022). Donor and recipient safety in human milk banking. Journal of paediatrics and child health, 58(9), 1629–1634. https://doi.org/10.1111/jpc.16066 | Wrong publication type |
| 9 | Thorley V. (2014) | Thorley V. (2014). Milk siblingship, religious and secular: History, applications, and implications for practice. Women and birth: journal of the Australian College of Midwives, 27(4), e16–e19. https://doi.org/10.1016/j.wombi.2014.09.003 | Wrong publication type |
| 10 | Simmer K. (2011) | Simmer K. (2011). The knowns and unknowns of human milk banking. Nestle Nutrition workshop series. Paediatric programme, 68, 49–64. https://doi.org/10.1159/000325659 | Wrong publication type |
| 11 | de Halleux et al. (2017) | de Halleux, V., Pieltain, C., Senterre, T., & Rigo, J. (2017). Use of donor milk in the neonatal intensive care unit. Seminars in fetal & neonatal medicine, 22(1), 23–29. https://doi.org/10.1016/j.siny.2016.08.003 | No full text |
| 12 | Garegrat et al. (2021) | Garegrat, R., Malshe, N., Suryawanshi, P., & Patnaik, S. K. (2021). Improving donor human milk collection in a hospital without a human milk bank: a quality improvement initiative at an urban tertiary-care teaching hospital. BMJ open quality, 10(Suppl 1), e001467. https://doi.org/10.1136/bmjoq-2021-001467 | Wrong publication type |
| 13 | Peregoy et al. (2022) | Peregoy, J. A., Pinheiro, G. M., Geraghty, S. R., Dickin, K. L., & Rasmussen, K. M. (2022). Human milk-sharing practices and infant-feeding behaviours: A comparison of donors and recipients. Maternal & child nutrition, 18(4), e13389. https://doi.org/10.1111/mcn.13389 | Wrong publication type |
| 14 | Pal et al. (2019) | Pal, A., Soontarapornchai, K., Noble, L., & Hand, I. (2019). Attitudes towards Donor Breast Milk in an Inner-City Population. International journal of pediatrics, 2019, 3847283. https://doi.org/10.1155/2019/3847283 | Wrong publication type |
| 15 | Kullmann et al. (2022) | Kullmann, K. C., Adams, A. C., & Feldman-Winter, L. (2022). Human Milk Sharing in the United States: A Scoping Review. Breastfeeding medicine: the official journal of the Academy of Breastfeeding Medicine, 17(9), 723–735. https://doi.org/10.1089/bfm.2022.0013 | No full text |
| 16 | Merlino-Barr et al. (2019) | Merlino-Barr, S., & Groh-Wargo, S. (2019). Donor Breast Milk for the Preterm Infant: Your Questions Answered! Neonatal network: NN, 38(1), 7–16. https://doi.org/10.1891/0730-0832.38.1.7 | Wrong publication type |
| 17 | Klotz et al. (2020) | Klotz, D., Jansen, S., Glanzmann, R., Haiden, N., Fuchs, H., & Gebauer, C. (2020). Donor human milk programs in German, Austrian and Swiss neonatal units - findings from an international survey. BMC pediatrics, 20(1), 235. https://doi.org/10.1186/s12887-020-02137-2 | Wrong publication type |
| 18 | Brownell et al. (2018) | Brownell, E. A., Matson, A. P., Smith, K. C., Moore, J. E., Esposito, P. A., Lussier, M. M., Lerer, T. J., & Hagadorn, J. I. (2018). Dose-response Relationship Between Donor Human Milk, Mother's Own Milk, Preterm Formula, and Neonatal Growth Outcomes. Journal of pediatric gastroenterology and nutrition, 67(1), 90–96. https://doi.org/10.1097/MPG.0000000000001959 | Wrong publication type |
| 19 | O'Sullivan et al (2016) | O'Sullivan, E. J., Geraghty, S. R., & Rasmussen, K. M. (2016). Informal Human Milk Sharing: A Qualitative Exploration of the Attitudes and Experiences of Mothers. Journal of human lactation: official journal of International Lactation Consultant Association, 32(3), 416–424. https://doi.org/10.1177/0890334416651067 | Wrong publication type |
| 20 | McGlothen-Bell et al. (2019) | McGlothen-Bell, K., Cleveland, L., & Pados, B. F. (2019). To Consent, or Not to Consent, That Is the Question: Ethical Issues of Informed Consent for the Use of Donor Human Milk in the NICU Setting. Advances in neonatal care: official journal of the National Association of Neonatal Nurses, 19(5), 371–375. https://doi.org/10.1097/ANC.0000000000000651 | Wrong publication type |
| 21 | Kair et al. (2017) | Kair, L. R., & Flaherman, V. J. (2017). Donor Milk or Formula: A Qualitative Study of Postpartum Mothers of Healthy Newborns. Journal of human lactation: official journal of International Lactation Consultant Association, 33(4), 710–716. https://doi.org/10.1177/0890334417716417 | Wrong publication type |
| 22 | Olonan-Jusi et al. (2021) | Olonan-Jusi, E., Zambrano, P. G., Duong, V. H., Anh, N. T. T., Aye, N. S. S., Chua, M. C., Kurniasari, H., Moe, Z. W., Ngerncham, S., Phuong, N. T. T., & Datu-Sanguyo, J. (2021). Human milk banks in the response to COVID-19: a statement of the regional human milk bank network for Southeast Asia and beyond. International breastfeeding journal, 16(1), 29. https://doi.org/10.1186/s13006-021-00376-2 | Wrong publication type |
| 23 | Edwards et al. (2012) | Edwards, T. M., & Spatz, D. L. (2012). Making the case for using donor human milk in vulnerable infants. Advances in neonatal care: official journal of the National Association of Neonatal Nurses, 12(5), 273–280. https://doi.org/10.1097/ANC.0b013e31825eb094 | Wrong publication type |
| 24 | BM, Reyes-Foster; SK, Carter; MS, Hinojosa; | Reyes-Foster, B. M., Carter, S. K., & Hinojosa, M. S. (2015). Milk sharing in practice: a descriptive analysis of peer breastmilk sharing. Breastfeeding medicine: the official journal of the Academy of Breastfeeding Medicine, 10(5), 263–269. https://doi.org/10.1089/bfm.2015.0009 | Wrong publication type |
| 25 | Updegrove K et al. (2013) | Updegrove K. (2013). Nonprofit Human Milk Banking in the United States. Journal of midwifery & women's health, 58(5), 502–596. https://doi.org/10.1111/j.1542-2011.2012.00267.x | Wrong publication type |
| 26 | Akre et al. (2011) | Akre, J. E., Gribble, K. D., & Minchin, M. (2011). Milk sharing from private practice to public pursuit. International breastfeeding journal, 6, 8. https://doi.org/10.1186/1746-4358-6-8 | Wrong publication type |
| 27 | Parker et al. (2013) | Parker, M. G., Barrero-Castillero, A., Corwin, B. K., Kavanagh, P. L., Belfort, M. B., & Wang, C. J. (2013). Pasteurized human donor milk use among US level 3 neonatal intensive care units. Journal of human lactation: official journal of International Lactation Consultant Association, 29(3), 381–389. https://doi.org/10.1177/0890334413492909 | Wrong publication type |
| 28 | Espina-Jerez et al. (2022) | Espina-Jerez, B., Romera-Álvarez, L., de Dios-Aguado, M., Cunha-Oliveira, A., Siles-Gonzalez, J., & Gómez-Cantarino, S. (2022). Wet Nurse or Milk Bank? Evolution in the Model of Human Lactation: New Challenges for the Islamic Population. International journal of environmental research and public health, 19(15), 9742. https://doi.org/10.3390/ijerph19159742 | Wrong publication type |
| 29 | Perrin et al. (2014) | Perrin, M. T., Goodell, L. S., Allen, J. C., & Fogleman, A. (2014). A mixed-methods observational study of human milk sharing communities on Facebook. Breastfeeding medicine: the official journal of the Academy of Breastfeeding Medicine, 9(3), 128–134. https://doi.org/10.1089/bfm.2013.0114 | Wrong publication type |
| 30 | Palmquist et al. (2014) | Palmquist, A. E., & Doehler, K. (2014). Contextualizing online human milk sharing structural factors and lactation disparity among middle income women in the U.S. Social science & medicine (1982), 122, 140–147. https://doi.org/10.1016/j.socscimed.2014.10.036 | No full text |
| 31 | Bocci et al. (2019) | Bocci, G., Kundisova, L., Pacini, V., Nante, N., & Alaimo, L. (2019). Generous breastfeeding: an observational retrospective study of milk donor's characteristics in the province of Siena, Italy. Annali di igiene: medicina preventiva e di comunita, 31(4), 316–325. https://doi.org/10.7416/ai.2019.2293 | No full text |
| 32 | Bortolozo et al. (2004) | Bortolozo, E. A., Tiboni, E. B., & Cândido, L. M. (2004). Leite humano processado em bancos de leite para o recém-nascido de baixo peso: análise nutricional e proposta de um novo complemento [Milk from human milk banks for low birthweight newborns: nutritional contents and supplementation]. Revista panamericana de salud publica = Pan American journal of public health, 16(3), 199–205. https://doi.org/10.1590/s1020-49892004000900007 | Wrong publication type |
| 33 | Keim et al. (2014) | Keim, S. A., McNamara, K. A., Dillon, C. E., Strafford, K., Ronau, R., McKenzie, L. B., & Geraghty, S. R. (2014). Breastmilk sharing awareness and participation among women in the Moms2Moms Study. Breastfeeding medicine: the official journal of the Academy of Breastfeeding Medicine, 9(8), 398–406. https://doi.org/10.1089/bfm.2014.0032 | No full text |
| 34 | Parker et al. (2019) | Parker, L. A., Cacho, N., Engelmann, C., Benedict, J., Wymer, S., Michael, W., & Neu, J. (2019). Consumption of Mother's Own Milk by Infants Born Extremely Preterm Following Implementation of a Donor Human Milk Program: A Retrospective Cohort Study. The Journal of pediatrics, 211, 33–38. https://doi.org/10.1016/j.jpeds.2019.03.040 | Wrong publication type |
| 35 | Tshamala et al. (2018) | Tshamala, D., Pelecanos, A., & Davies, M. W. (2018). Factors associated with infants receiving their mother's own breast milk on discharge from hospital in a unit where pasteurised donor human milk is available. Journal of paediatrics and child health, 54(9), 1016–1022. https://doi.org/10.1111/jpc.14062 | Wrong publication type |
| 36 | Arnold LD (2008) | Arnold L. D. (2008). U.S. health policy and access to banked donor human milk. Breastfeeding medicine: the official journal of the Academy of Breastfeeding Medicine, 3(4), 221–229. https://doi.org/10.1089/bfm.2007.0037 | No full text |
| 37 | Alves et al. (2016) | Alves, E., Magano, R., Amorim, M., Nogueira, C., & Silva, S. (2016). Factors Influencing Parent Reports of Facilitators and Barriers to Human Milk Supply in Neonatal Intensive Care Units. Journal of human lactation: official journal of International Lactation Consultant Association, 32(4), 695–703. https://doi.org/10.1177/0890334416664071 | Wrong publication type |
| 38 | Miller et al. (2018) | Miller, A. R., Fenstermacher, K., & Buchko, B. L. (2018). Going Along with It: Neonatal Intensive Care Nurses' Views of a Donor Milk Practice Change. MCN. The American journal of maternal child nursing, 43(5), 285–290. https://doi.org/10.1097/NMC.0000000000000454 | Wrong publication type |
| 39 | Kamholz et al. (2012) | Kamholz, K. L., Parker, M. G., & Philipp, B. L. (2012). Implementing change: steps to initiate a human donor milk program in a US Level III NICU. Journal of human lactation: official journal of International Lactation Consultant Association, 28(2), 128–131. https://doi.org/10.1177/0890334412438962 | Wrong publication type |
| 40 | El-Khuffash et al. (2012) | El-Khuffash, A., & Unger, S. (2012). The concept of milk kinship in Islam: issues raised when offering preterm infants of Muslim family’s donor human milk. Journal of human lactation: official journal of International Lactation Consultant Association, 28(2), 125–127. https://doi.org/10.1177/0890334411434803 | Wrong publication type |
| 41 | Thorley V (2008) | Thorley V. (2008). Breasts for hire and shared breastfeeding: wet nursing and cross feeding in Australia, 1900-2000. Health and history, 10(1), 88–109. | Wrong publication type |
| 42 | Kültürsay et al. (2018) | Kültürsay, N., Bilgen, H., & Türkyılmaz, C. (2018). Turkish Neonatal Society guideline on enteral feeding of the preterm infant. Turk pediatri arsivi, 53(Suppl 1), S109–S118. https://doi.org/10.5152/TurkPediatriArs.2018.01811 | Wrong publication type |
| 43 | Mgongo et al. (2019) | Mgongo, M., Hussein, T. H., Stray-Pedersen, B., Vangen, S., Msuya, S. E., & Wandel, M. (2019). Facilitators and Barriers to Breastfeeding and Exclusive Breastfeeding in Kilimanjaro Region, Tanzania: A Qualitative Study. International journal of pediatrics, 2019, 8651010. https://doi.org/10.1155/2019/8651010 | Wrong publication type |
| 44 | Paramashanti et al. (2022) | Paramashanti, B. A., Dibley, M. J., Huda, T. M., & Alam, A. (2022). Breastfeeding perceptions and exclusive breastfeeding practices: A qualitative comparative study in rural and urban Central Java, Indonesia. Appetite, 170, 105907. https://doi.org/10.1016/j.appet.2021.105907 | Wrong publication type |
| 45 | Picaud JC (2022) | Picaud J. C. (2022). Review highlights the importance of donor human milk being available for very low birth weight infants. Acta paediatrica (Oslo, Norway: 1992), 111(6), 1127–1133. https://doi.org/10.1111/apa.16296 | Wrong publication type |
| 46 | Boundy et al. (2022) | Boundy, E. O., Anstey, E. H., & Nelson, J. M. (2022). Donor Human Milk Use in Advanced Neonatal Care Units - United States, 2020. MMWR. Morbidity and mortality weekly report, 71(33), 1037–1041. https://doi.org/10.15585/mmwr.mm7133a1 | Wrong publication type |
| 47 | Lee ML (2006) | Lee M. L. (2006). The design and analysis of studies in premature infants using human donor milk or preterm formula as primary nutrition: a critique of Schanler et al. Breastfeeding medicine: the official journal of the Academy of Breastfeeding Medicine, 1(2), 88–93. https://doi.org/10.1089/bfm.2006.1.88 | Wrong publication type |
| 48 | Ahmed et al. (2022) | Mohammed A.M. Ahmed, Charles Patrick Namisi, Nakibuuka Viktoria Kirabira et al. Acceptability to donate human breast milk among post-natal mothers at St Francis hospital Nsambya Uganda: A mixed method study, 13 May 2022, PREPRINT (Version 1) available at Research Square [https://doi.org/10.21203/rs.3.rs-1631314/v1] | Wrong publication type |
| 49 | Brandstetter et al. (2018) | Brandstetter, S., Mansen, K., DeMarchis, A., Nguyen Quyhn, N., Engmann, C., & Israel-Ballard, K. (2018). A Decision Tree for Donor Human Milk: An Example Tool to Protect, Promote, and Support Breastfeeding. Frontiers in pediatrics, 6, 324. https://doi.org/10.3389/fped.2018.00324 | Wrong publication type |
| 50 | Ghaly 2012 | Ghaly M. (2012). Milk banks through the lens of Muslim scholars: one text in two contexts. Bioethics, 26(3), 117–127. https://doi.org/10.1111/j.1467-8519.2010.01844.x | Wrong publication type |
| 51 | Bai et al. (2021) | Bai, Y., & Kuscin, J. (2021). The Current State of Donor Human Milk Use and Practice. Journal of midwifery & women's health, 66(4), 478–485. https://doi.org/10.1111/jmwh.13244 | Wrong publication type |
| 52 | Khalil et al. (2016) | Khalil, A., Buffin, R., Sanlaville, D., & Picaud, J. C. (2016). Milk kinship is not an obstacle to using donor human milk to feed preterm infants in Muslim countries. Acta paediatrica (Oslo, Norway : 1992), 105(5), 462–467. https://doi.org/10.1111/apa.13308 | Wrong publication type |
| 53 | Doshmangir et al. (2019) | Doshmangir, L., Naghshi, M., & Khabiri, R. (2019). Factors Influencing Donations to Human Milk Bank: A Systematic Review of Facilitators and Barriers. Breastfeeding medicine : the official journal of the Academy of Breastfeeding Medicine, 14(5), 298–306. https://doi.org/10.1089/bfm.2019.0002 | Wrong publication type |
| 54 | Santos et al. (2022) | Gutierrez Dos Santos, B., & Perrin, M. T. (2022). What is known about human milk bank donors around the world: a systematic scoping review. Public health nutrition, 25(2), 312–322. https://doi.org/10.1017/S1368980021003979 | Wrong publication type |
| 55 | Clouthier et al. (2019) | Clouthier, N., Ulrich, C., & Hartman, D. B. (2019). Acceptability of Donor Human Milk in Muslim Populations in Canada. Canadian journal of dietetic practice and research : a publication of Dietitians of Canada = Revue canadienne de la pratique et de la recherche en dietetique : une publication des Dietetistes du Canada, 80(4), 186–189. https://doi.org/10.3148/cjdpr-2019-006 | Wrong publication type |
| 56 | Ozdemir et al. (2015) | Ozdemir, R., Ak, M., Karatas, M., Ozer, A., Dogan, D. G., & Karadag, A. (2015). Human milk banking and milk kinship: perspectives of religious officers in a Muslim country. *Journal of perinatology : official journal of the California Perinatal Association*, *35*(2), 137–141. https://doi.org/10.1038/jp.2014.177 | Wrong publication type |
